# Posttraumatic Subepineural Hematoma of the Median Nerve Diagnosed With Ultrasound Imaging

**DOI:** 10.7759/cureus.83270

**Published:** 2025-04-30

**Authors:** Alena Nixon, Adeola Ajayi, Steve K Lee, Ogonna K Nwawka

**Affiliations:** 1 Radiology, Staten Island University Hospital, Staten Island, USA; 2 Radiology, Drexel University College of Medicine, Philadelphia, USA; 3 Orthopedics, Hospital for Special Surgery, New York, USA; 4 Radiology and Imaging, Hospital for Special Surgery, New York, USA

**Keywords:** hematoma, median nerve, neuropathy, peripheral nerve trauma, ultrasound

## Abstract

This case report demonstrates the utility of ultrasound (US) in the diagnosis, characterization, and follow-up of an intraneural median nerve hematoma following peripherally inserted central catheter (PICC) placement. A patient presenting with neuropathy in the median nerve distribution was evaluated in the US clinic. US successfully identified an intraneural hematoma of the median nerve, characterized it as subepineural, and detected its incomplete resolution during follow-up imaging. This case highlights the value of the US as a first-line modality for the evaluation of median nerve hematoma in patients exhibiting neuropathy following PICC placement. On US, a subepineural intraneural hematoma will cause eccentric displacement of and mass effect on nerve fascicles, with both the hematoma and fascicles encased within the epineurium.

## Introduction

Intraneural hematomas are an uncommon phenomenon, typically occurring in the setting of anticoagulation, trauma, or neoplasm [1]. Patients with this condition often present with neurological defects in the distribution of the affected nerve [1]. Symptoms may persist long after the initial injury and may be debilitating. As such, timely diagnosis and treatment may be critical for corrective management of this condition. Limited available literature, including radiologic evaluation, has largely depicted magnetic resonance imaging (MRI) findings of intraneural hematoma [1-4], typically describing masses within nerves showing characteristics suggesting internal blood products. However, sonography is a well-established modality for the evaluation of peripheral nerve abnormalities [5]. This report presents a case of post-traumatic intraneural hematoma of the median nerve primarily evaluated with high-resolution ultrasound (US) imaging.

## Case presentation

**Figure 1:**
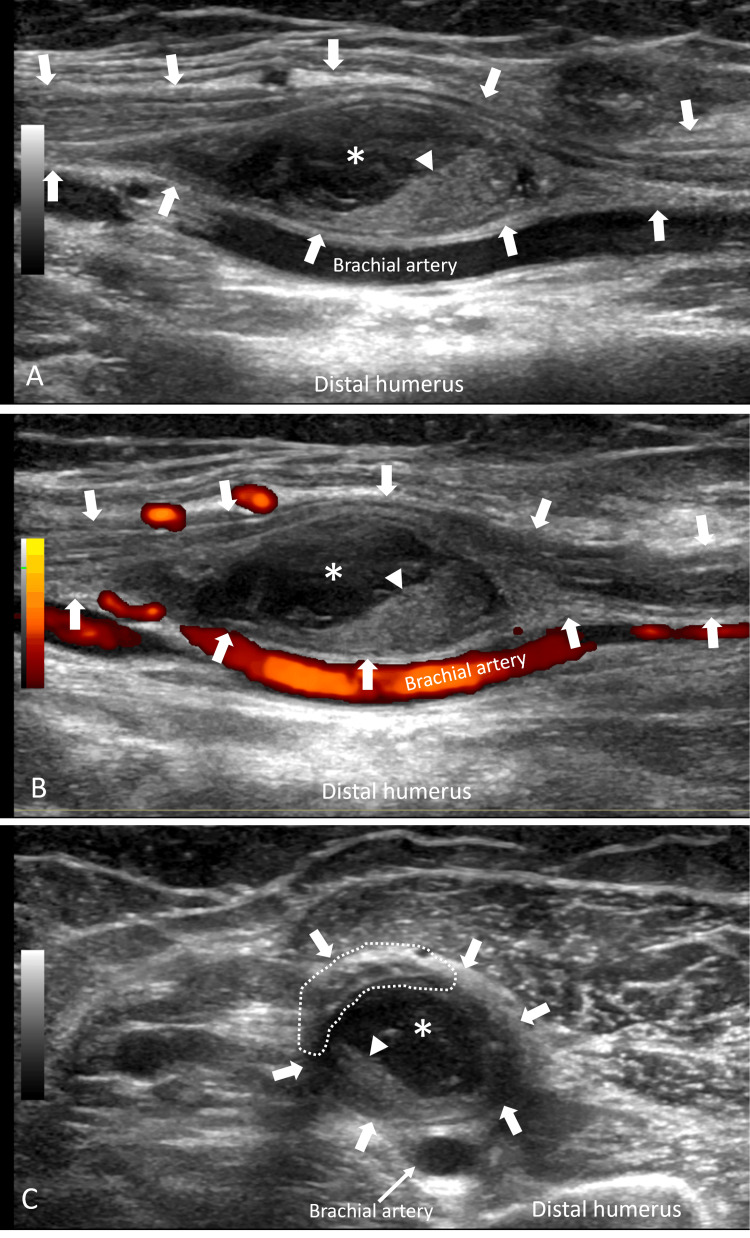
Median nerve ultrasound imaging in a 67-year-old woman with median neuropathy symptoms 2.5 months after traumatic PICC placement PICC: peripherally inserted central catheter Longitudinal grayscale (A) and power Doppler (B) US images and transverse grayscale (C) US image demonstrate a fusiform complex mass (*) encased within the median nerve epineurium (block arrows). The mass contains low-level echoes as well as echogenic eccentric tissue (arrowhead), features suggesting hematoma with internally retractile clot. The hematoma is encased in the epineurium but eccentrically displaces and compresses the nerve fascicles (dotted outline), consistent with subepineural position. Note the clear delineation of fascicular anatomy on longitudinal and transverse imaging

A 67-year-old woman presented at the US clinic with left-hand numbness and weakness for two and a half months. Three months prior, the patient was hospitalized after a motor vehicle accident resulting in polytrauma with multiple extremity fractures requiring hospitalization. Of note, the patient was not anticoagulated during or before admission, and there was no fracture in the left arm. Prior to discharge, left upper arm peripherally inserted central catheter (PICC) placement required for long-term antibiotics produced a large hematoma on the arm. The patient noticed concomitant numbness and tingling in the thumb, index, and middle fingers, which progressed to weakness of left thumb and index finger flexion. These symptoms persisted with her presentation at the US clinic. Sonographic evaluation performed using a 15 MHz transducer (GE Healthcare, Waukesha, WI) demonstrated fusiform enlargement of the median nerve adjacent to the brachial artery and vein at the distal upper arm, caused by a large internally complex subepineural mass (Figure 1).

**Figure 2:**
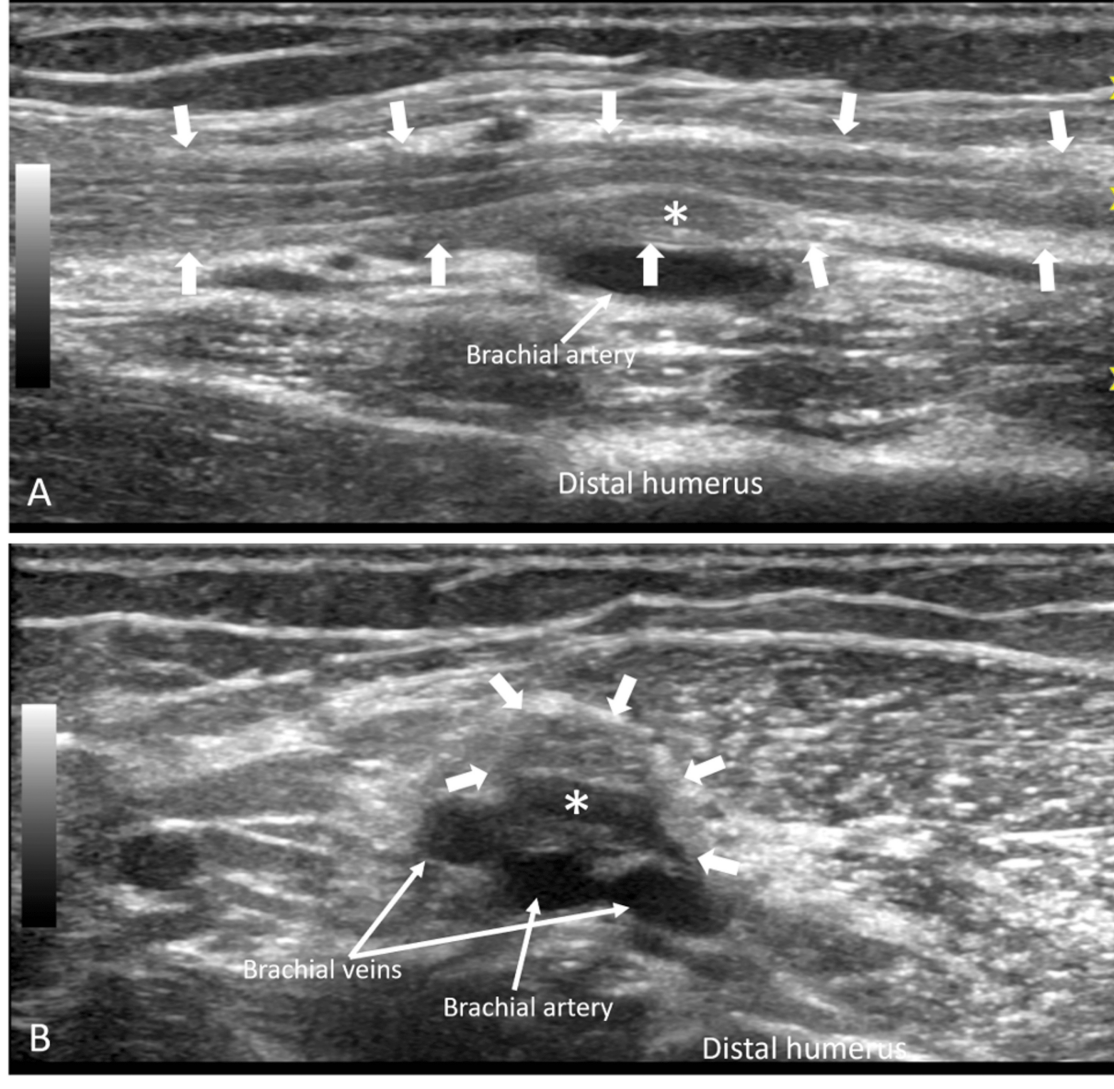
Follow-up median nerve ultrasound imaging in a 67-year-old woman with residual median neuropathy 4.5 months after traumatic PICC placement PICC: peripherally inserted central catheter Longitudinal (A) and transverse (B) grayscale US images demonstrate a marked interval decrease in size of the subepineural hematoma (*) encased within the median nerve epineurium (block arrows). The mass exerts much less mass effect on the fascicles, which are clearly distinguished

This mass eccentrically displaced and severely impinged on the median nerve fascicles. Imaging features were consistent with a subepineural hematoma. Electrodiagnostic evaluation two weeks later showed left median neuropathy localizing to above the elbow, resulting in complete denervation to all muscles innervated by the median and anterior interosseous nerves, and no evidence of axonal regeneration. Two months after their initial US clinic presentation, follow-up US imaging showed decreased hematoma size (Figure 2).

However, as the patient showed no symptomatic improvement in weakness, neurolysis with hematoma evacuation was performed shortly after. Intraoperative findings confirmed subepineural hematoma but also severe perineural and intraneural scarring. At their two-week postoperative check, the patient regained strength in their index finger and improved sensation in the thumb and index finger. At five months postop, strength was almost normal, but there was a residual sensation decrease. At 10 months postop, sensation further improved but was still not completely normal. The patient was counseled that sensation may never fully recover, but the patient was overall content with their recovery at this point.

## Discussion

The present case describes the diagnosis and follow-up of a post-traumatic subepineural hematoma within the median nerve using US imaging. Imaging demonstrated clear fascicular mass effect, and subsequent neurolysis findings highlighted the potential complications of chronic post-traumatic intraneural hematoma.

Classification of intraneural hematomas has been established based on their location within the nerve, and management has been proposed based on this classification [1]. Hematoma in the subepineural location will cause eccentric displacement of nerve fascicles, which can compromise nerve function, as in the current case. Clinically available high-resolution US transducers can resolve epineural and fascicular anatomy and can readily diagnose and characterize nerve trauma [5]. As such, the US is well-suited to evaluate for fascicular level injury, particularly affecting the median nerve in the upper extremity.

Prior case reports reporting MRI features have shown high signal masses with features of blood products replacing nerve tissue [1-4]. However, fascicular resolution was not described or depicted in these cases. In contrast to MRI, US is less susceptible to motion and metallic artifacts and offers greater spatial resolution [5]. Electrodiagnostic evaluation in this patient clearly detected nerve injury localized to the distal upper arm. US findings provided needed characterization of this injury so the appropriate management plan could be generated.

No consensus yet exists on the most appropriate management for intraneural hematoma [1]. Hematomas may spontaneously resolve or may be surgically decompressed with techniques based on the location of the hematoma [1]. Surgical reasoning for decompression is largely to prevent irreversible nerve damage [1,3]. Even though hematomas can resolve, there may be residual intraneural scarring, which will require neurolysis, as in the present case. This patient’s hematoma was much smaller on follow-up imaging, but their symptoms persisted, attributed to residual scar tissue that formed due to trauma. The delay from the patient’s initial injury to the time of presentation may have contributed to the degree of scar formation, and it is unclear whether more rapid hematoma evacuation could have resulted in less scarring. However, a more timely evacuation of hematoma has been associated with faster functional recovery and decreased axonal damage in a rat model [6]. Clinically, one can expect neuropathy to persist for many months, even with treatment [1-7].

## Conclusions

This diagnosis of intraneural hematoma is rare, but the inciting procedure of PICC placement is quite common. Given the proximity of the median nerve to the brachial veins in the distal upper arm, penetrating nerve injury, including hematoma formation, should be suspected if median neuropathy occurs after PICC placement. In contrast to prior case examples using MRI, this report demonstrates the utility of US in diagnosing intraneural hematoma, and its ability to characterize the hematoma location offers clinicians information critical to appropriate management. If symptoms of nerve dysfunction occur post PICC placement, US should be considered a first-line modality in evaluating for nerve injury.
